# Geometric Abstraction and Parametric Simplification of Camphor Leaf Venation for Biomimetic Functional Surface Texture Design

**DOI:** 10.3390/biomimetics11090661

**Published:** 2026-09-15

**Authors:** Zhengzhi Yang, Yao Yao, Qianqian Wang

**Affiliations:** 1 Jiangxi Polytechnic University, Jiujiang 332000, China; 2 De Institute of Creative Arts and Design, UCSI University, Kuala Lumpur 56000, Malaysia

**Keywords:** biomimetic design, leaf venation, geometric simplification, functional surface texture, anti-slip, tactile feedback, parametric abstraction

## Abstract

No standardized workflow currently exists for translating leaf venation—a natural conduction network—into functional surface texture for product design. Taking the basal actinodromous venation of *Cinnamomum camphora* (camphor tree) leaves as its object of study, this paper proposes a parametric workflow spanning standardized specimen collection, high-resolution imaging, manual layered vector tracing, and a three-stage geometric simplification algorithm. Simplification performance is quantified through three metrics: curve simplification ratio (CSR), topology retention index (TRI), and area-coverage ratio (ACR). To assess how these simplified textures perform in practice, textures from all three stages were fabricated as silicone grip sleeves and evaluated through wet friction testing and tactile direction-recognition experiments. The three stages performed differently. Stage 1 yielded the highest wet friction coefficient. Stage 2 offered the best overall balance among friction enhancement, direction recognizability, and manufacturing feasibility. Stage 3, with the simplest geometry, proved easiest to fabricate. Differences in CSR and ACR across stages were statistically significant (*p* < 0.001), while TRI is reported separately as a descriptive reference measure. Together, the workflow offers a complete procedural framework and a quantitative basis for designing functional textures in biomimetic grip interfaces.

## 1. Introduction

Biological systems offer engineering and design a range of structural prototypes, among them the venation networks of leaves [1,2]. As the vascular network embedded within a leaf, venation transports water, nutrients, and photosynthetic products [3,4], with a geometry that approximates optimal transport topology [5,6]. Leaf vein geometry affects both transport efficiency and mechanical performance. One study [7] measured the angle between secondary and primary veins at approximately 50°, demonstrating through energy-minimization principles that this angle minimizes the bending energy of the venation. Biomimetic panel structures designed on this basis—using a 50° branching angle—showed flexural resistance more than 60% higher than structures built with a 90° angle. This finding highlights branching angle as an important geometric parameter associated with the mechanical performance of leaf venation, motivating the empirical determination of angular regularization targets undertaken in the present study (Section 2.4). The core methodology of biomimicry is to extract structural principles from biological systems and apply them to engineering or design problems [8,9,10].

Biomimicry has followed two distinct trajectories. In visually oriented biomimetic design practice [11], leaf venation serves as a formal reference, and the design process rests on the designer’s subjective judgment rather than any standardized procedure or quantifiable intermediate variable. Function-oriented research on biomimetic venation, by contrast, has developed a well-defined methodological framework: leaf-inspired microfluidic devices [12,13], fog-harvesting surfaces [14,15], and structural support frameworks [16] are all grounded in physical models and quantitative metrics. Computational generative models offer a partial bridge between these two directions [17], yet their parameters are developmental-biology variables rather than geometric ones, and the resulting structures require extensive manual post-processing before they become usable—biological fidelity and the controllability that design demands tend to trade off against each other. This tension is the principal reason the present study adopts manual layered tracing.

Among the work most directly relevant to this study, several efforts have tuned surface functional performance by adjusting textural geometric parameters—spacing, height, and orientation [18,19]—though their design prototypes remain largely regular geometric patterns, without incorporating the hierarchical structural features of biological form. Existing approaches to leaf vein simplification and abstraction, meanwhile, are oriented mainly toward biostatistics or visualization rather than manufacturing-oriented generation of functional textures. What distinguishes the present study is its use of the basal actinodromous venation of *Cinnamomum camphora* as a biological prototype: a quantifiable geometric simplification algorithm generates a morphological lineage, and functional experiments then establish the quantitative relationship between simplification level and anti-slip performance.

The basal actinodromous venation of *Cinnamomum camphora* was selected as the biological prototype for this study because its three primary veins exhibit a consistent geometric relationship with secondary veins—unlike pinnate or palmate venation, where branching angles vary more widely across the leaf surface. Native to southern China, Taiwan, Japan, and Southeast Asia [20], the species displays a characteristic basal three-veined (actinodromous) leaf form, in which three primary veins emerge from the base of the petiole and curve outward toward the leaf tip and margin—a configuration that remains highly stable within the species. East Asia preserves rich relict plant diversity, and long-term stable climatic refugia in the region have been identified [21]. Population-level studies further report phenotypic variation among individual old camphor trees [22]. This combination satisfies two requirements for the experimental object of this study: a structural prototype governed by parameterizable geometric regularities, and a structure that retains morphological flexibility within its natural range of variation—allowing the simplification algorithm’s response to biological variation to be tested.

The application scenario for this study is friction-enhancing texture design for hand-tool grips under wet conditions. Tool slippage ranks among the leading causes of hand-related occupational injury. Under wet conditions, surface texture must serve two functions simultaneously: enhancing friction and supporting directional tactile recognition. Because this scenario demands that a texture satisfy both visual identifiability and physical performance criteria—slip resistance and tactile feedback alike—it offers a suitable vehicle for functional validation.

To address these two gaps—the absence of quantifiable intermediate variables in the design process, and the disconnect between generation and validation—this study makes three methodological contributions. First, three parametric metrics (CSR, TRI, and ACR) establish a quantitative link between form and function. Second, a three-stage simplification algorithm constructs a continuous morphological lineage, from which designers can select the abstraction level appropriate to a given application. Third, the simplified textures were fabricated into physical prototypes, yielding experimental data—through wet friction and tactile-recognition testing—on the relationship between geometric metrics and functional performance.

**Figure 1:**
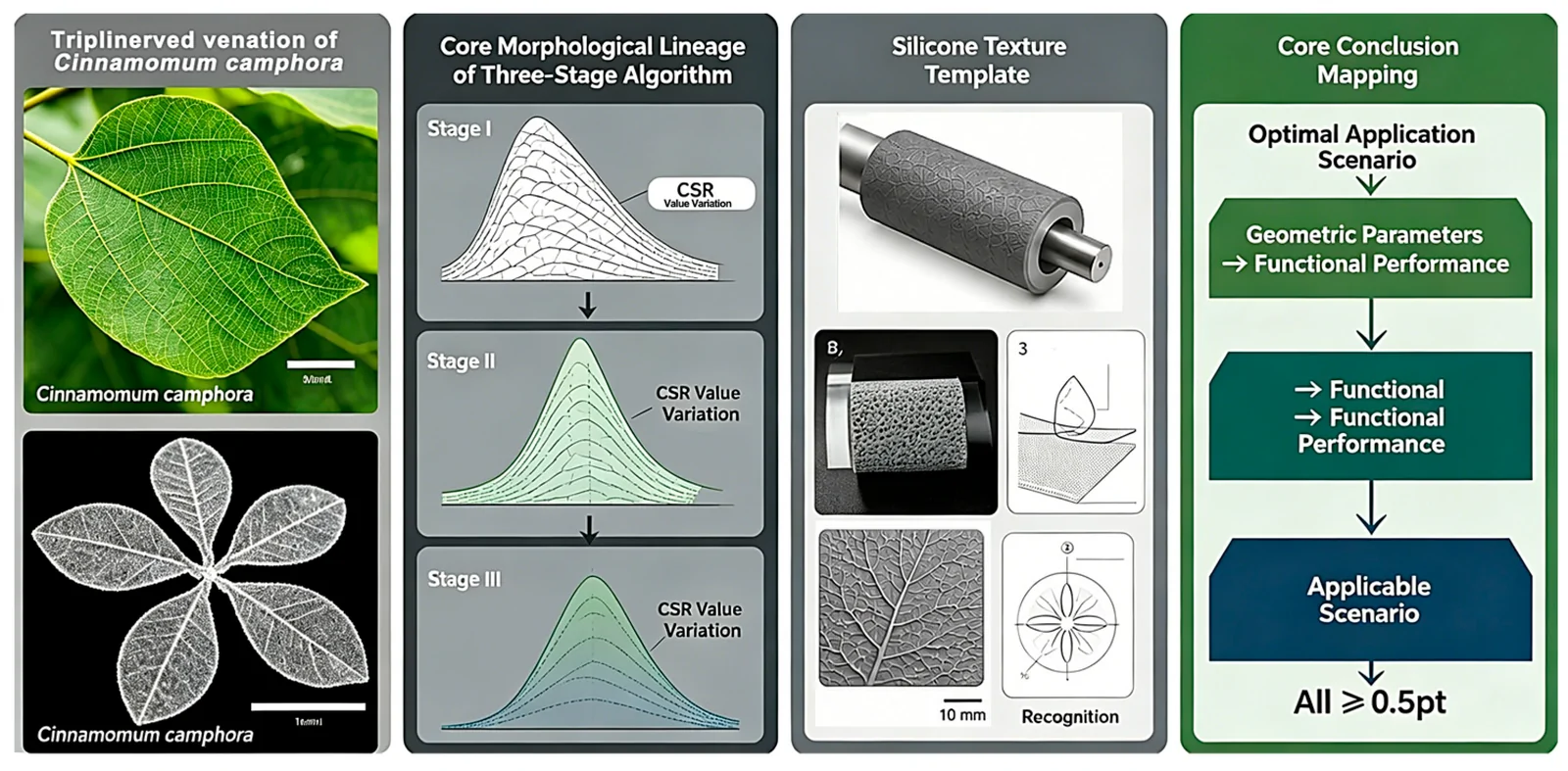
Biomimetic functional texture design workflow: *Cinnamomum camphora* leaf venation abstraction and simplification.

This study sets out three objectives: to develop a standardized workflow for leaf specimen collection, imaging, and layered tracing; to propose a three-stage parametric geometric simplification algorithm for constructing a morphological lineage; and to conduct proof-of-concept functional testing through friction and tactile-recognition experiments. The aim is a reproducible, quantifiable, and manufacturable design process (refer to Figure 1) for biomimetic functional textures—not a market-ready industrial product. Accordingly, this work is best characterized as a design-driven proof-of-concept in biomimicry: it examines the feasibility of translating biological morphology into manufacturable functional texture, and stops short of full friction-mechanism modeling or industrial engineering optimization.

## 2. Materials and Methods

### 2.1. Specimen Collection and Study Sites

**Figure 2:**
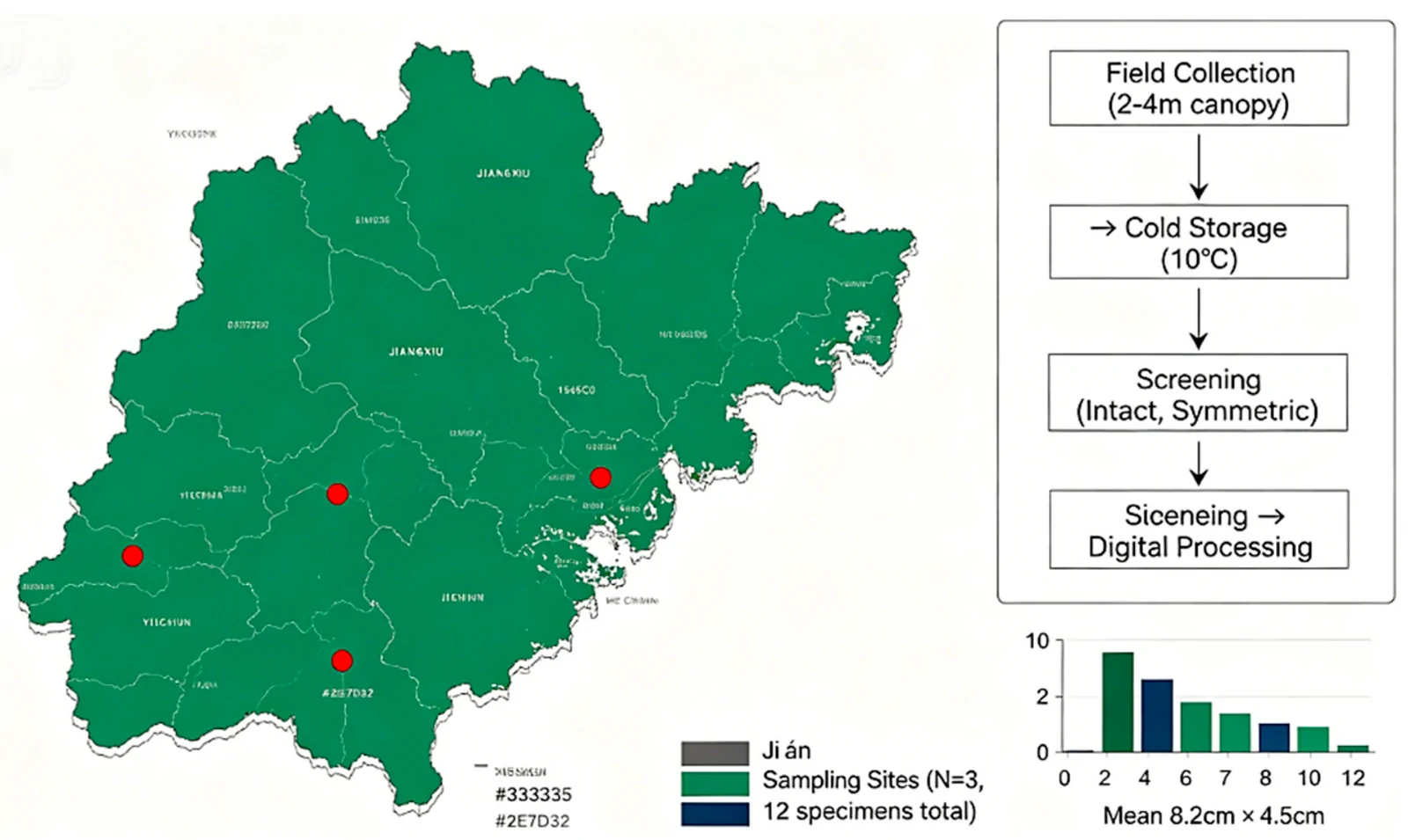
Schematic diagram of study area and specimen sampling scheme.

Camphor leaf specimens were collected between May and June 2025 from three old-growth *Cinnamomum camphora* stands in Ji’an and Yichun, Jiangxi Province, China (27°06′–28°42′ N, 114°30′–115°54′ E). The region (refer to Figure 2) has a subtropical humid monsoon climate (Köppen Cfa), with a mean annual temperature of 17–19 °C and annual precipitation of 1400–1900 mm.

Sites were selected against three criteria: each had to contain at least five mature camphor trees over 100 years old; each was located adjacent to a traditional tea-growing area (relevant to cultural design research outside the scope of this study); and leaves showed minimal anthropogenic disturbance—pruning, pollution exposure, and the like.

At each site, five to eight fully expanded, undamaged sun leaves were collected from the mid-to-lower canopy (2–4 m height) using a pole pruner, yielding 21 initial specimens. Nine were excluded for defects, including insect damage, mechanical injury, and bilateral asymmetry exceeding 20% of maximum leaf width; among the excluded specimens, the defects were mainly marginal tearing or localized insect damage, with no systematic morphological bias observed. Leaves were immediately pressed between moist filter paper, sealed in polyethylene bags, and stored at approximately 10 °C to minimize dehydration and morphological distortion. Twelve leaves were ultimately selected for digital processing and subsequent experiments.

### 2.2. Digital Imaging and Preprocessing

Imaging was completed within 24 h of specimen collection using a Sony mirrorless camera (Sony Corporation, Tokyo, Japan.)fitted with a Sony FE 90 mm f/2.8 macro lens (maximum magnification 1.0×). The camera was mounted on a Kaiser RS 1 copy stand, with a dual-axis bubble level ensuring the sensor plane was parallel to the specimen. The working distance was fixed at 45.0 cm, yielding a field of view of roughly 15 × 10 cm and a spatial resolution of approximately 55 pixels/mm.

Illumination was provided by two Godox SL60W LED panels (Godox Photo Equipment Co., Ltd., Shenzhen, China) (5600 K, CRI 95+), positioned symmetrically at a 45° angle to suppress specular reflection. Each specimen was photographed alongside a Kodak 18% gray card, an X-Rite ColorChecker Passport, and a millimeter scale bar. RAW images (ARW, 14-bit, 8192 × 5464 pixels) were captured at ISO 100, f/8.0, with the in-camera white balance set to 5600 K.

Post-processing was carried out in Adobe Lightroom Classic v13.2: white balance was fine-tuned against the neutral gray patch of the color card, and distortion and chromatic aberration were corrected using the manufacturer’s lens profile. Final images were exported as 16-bit uncompressed TIFF files at 300 PPI. All preprocessing steps were batch-logged to ensure the workflow’s reproducibility.

### 2.3. Manual Layered Vector Tracing

**Figure 3:**
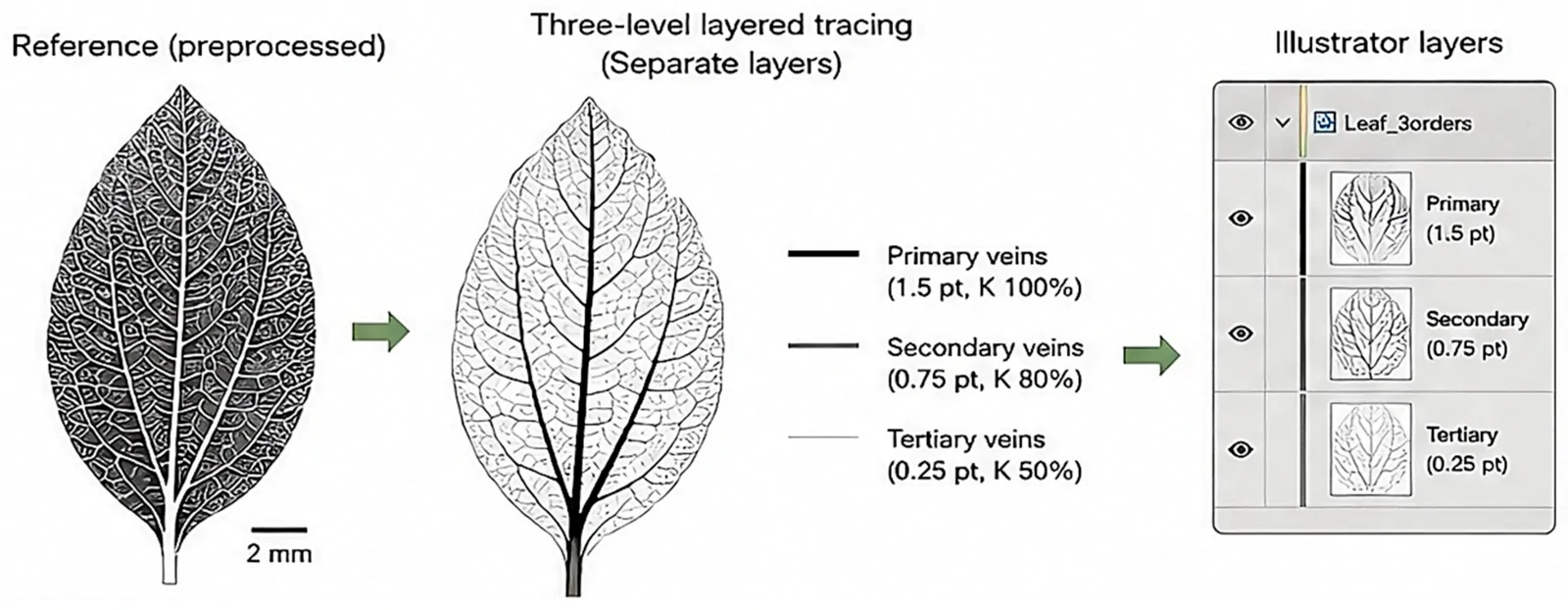
Manual layered tracing in Adobe Illustrator.

Manual tracing was chosen over automated segmentation methods (refer to Figure 3)—threshold-based skeletonization or deep learning [23,24]—for reasons detailed in Section 4.1. Tracing was performed on a Wacom Intuos Pro M tablet in Adobe Illustrator 2024 (v28.3).

Based on the structural features of the basal actinodromous venation in camphor leaves, the venation network was divided into three hierarchical layers by thickness, branching order, and visual prominence: primary veins—the three dominant veins emerging from the petiole base—rendered as 1.5 pt black lines (CMYK 0/0/0/100); secondary veins, branching from the primary veins at 45–60°, rendered as 0.75 pt dark gray lines (CMYK 0/0/0/80); and tertiary veins, the fine reticulate network filling the interveinal areas, rendered as 0.25 pt light gray lines (CMYK 0/0/0/50). Each layer was assigned to a separately named Illustrator layer to allow selective simplification downstream.

Tracing was performed by a single operator following a written protocol, after standardized training on three pilot specimens. Specimen CAM-07 was independently retraced by a second operator for inter-rater reliability assessment; the check showed high overlap in primary and secondary vein paths, with local deviations consistently below a 0.5 mm tolerance threshold. A second specimen, CAM-11, was also double-traced to further confirm the robustness of the results.

Accuracy was assessed against three metrics: mean nearest-neighbor distance (MNND) at branch junctions, Hausdorff distance between vector networks, and per-layer classification accuracy for primary, secondary, and tertiary veins. Cohen’s κ was additionally used to quantify agreement in layer classification.

Manual tracing is time-consuming, but it yields a vector structure with clear hierarchy and visual control—one compatible with downstream computational analysis and extensible to semi-automated workflows in future work.

### 2.4. Three-Stage Geometric Simplification Algorithm

This study proposes a three-stage iterative simplification algorithm that converts the biomimetic vector tracing into a morphological lineage suited to different applications, including anti-slip surface texture design for hand-tool grips. The algorithm was developed under three constraints. For legibility, the simplified pattern had to remain recognizable as leaf venation at a minimum size of 10 × 10 mm. For reproducibility, textural features had to remain stable across common production processes—offset printing, hot-stamping, and 72 PPI screen display among them. For systemic continuity, visual style had to remain coherent across simplification stages, so that designers could select the abstraction level appropriate to a given application.

To inform the angular regularization parameters used in the simplification algorithm, nine camphor leaf specimens—drawn from the same three old-growth stands described in Section 2.1—were selected for direct measurement of the angles between primary and secondary veins. A protractor-based angle tool was used to measure the angles among the three primary veins and all secondary vein emergence angles for each specimen, yielding 134 secondary-vein angle records in total.

The core parameters of the algorithm were not arbitrarily set but derived from pilot testing and statistical analysis, as detailed below.

(1) Chord-height ratio curvature threshold of 0.05

Three additional independent specimens were used for preliminary testing across three candidate thresholds: 0.03, 0.05, and 0.07. A threshold of 0.03 over-preserved minor curvature variation, resulting in insufficient geometric regularization; 0.07, conversely, smoothed the natural venation curvature excessively, reducing the topology retention index (TRI) by more than 8%. The 0.05 threshold performed most consistently—retaining essential curvature characteristics while achieving effective geometric regularization and preserving suitability for hand-tool grip texturing.

(2) Angular regularization tolerance of ±5°

Based on measurements from 134 secondary-vein branch points across nine specimens, the secondary-vein emergence angle relative to the primary vein averaged 56.92° ± 4.43° (specimen-level mean ± SD). This value sits close to the 45°/60° regularization target range adopted in this study, and its relatively small standard deviation suggests a degree of natural stability in secondary-vein branching angle within the species—providing empirical support for adopting 45°/60° as the geometric regularization benchmark. This value lies between the two adopted targets, indicating that the 45°/60° scheme is not intended to reproduce the measured mean directly. Instead, the two angles serve as geometric regularization benchmarks that convert naturally variable venation geometry into simplified and manufacturable design parameters, rather than biological averages.

Preliminary testing compared three tolerance levels: ±2°, ±5°, and ±10°. A ±2° tolerance proved too strict, leaving many branches unregularized; ±10° was too permissive, altering original angles excessively and eroding species-specific characteristics. The ±5° tolerance struck the best balance between regularization effectiveness and morphological fidelity. This tolerance was accordingly adopted.

(3) Vein segment length threshold of 2 mm

The 2 mm threshold follows industrial standards for offset printing and hot-stamping, processes in which segments shorter than 2 mm commonly break or blur and are therefore treated as artifacts or non-functional. This filtering step affected only 0–2 segments per specimen across the 12 specimens (median: 1), with negligible impact on overall topological structure.

The specific operations at each simplification stage follow Table 1, with the rationale for each key parameter detailed in (1)–(3) above.

### 2.5. Quantitative Assessment of Simplification Degree

For each simplification stage, this study defines and computes three metrics (*n* = 12) to quantify the transformation from biological prototype to geometric abstraction and to provide a quantitative basis for selecting anti-slip textures.

The three metrics are defined as follows.

Curve Simplification Ratio (CSR)

$$CSR=\frac{N_{\mathrm{simplified}}}{N_{\mathrm{original}}}$$

Here, N denotes the total number of anchor points. A lower CSR indicates a higher degree of simplification and lower manufacturing complexity, with different CSR levels corresponding to different production-cost requirements for hand-tool grip texturing. To support the reproducibility of CSR as a geometric complexity metric, anchor-point generation was standardized as follows: (1) all vector tracings were performed by a single trained operator following a written protocol; (2) the tracing protocol was validated through double-tracing of two specimens, confirming inter-operator consistency; and (3) the vectorization procedure in Adobe Illustrator used consistent curve-fitting tolerances and anchor-placement rules across all specimens, with anchors placed only at curvature-change points, branch nodes, and endpoints. We acknowledge that CSR may still carry some degree of operator dependence; automated or semi-automated vectorization is identified in Section 4.4 as a future direction for improving the reproducibility of this metric.

Topology Retention Index (TRI)

$$TRI=\frac{B_{\mathrm{simplified}}}{B_{\mathrm{original}}}$$

TRI is defined as B_simplified/B_original, where B denotes the branching-structure elements of the original network retained in the simplified version—branch junctions and their relative spatial positions. In Stages 1 and 2, the secondary vein segments corresponding to branch nodes are retained alongside the nodes themselves, so TRI at these stages reflects the completeness of branching connectivity. Because Stage 3 retains only the primary veins, with secondary and higher-order branches removed entirely, its statistical basis for TRI differs fundamentally from that of the earlier stages. Accordingly, the Stage 3 value serves only as a descriptive reference for residual topological information and should not be used for cross-stage quantitative comparison or included in statistical testing.

Area Coverage Ratio (ACR)

$$ACR=\frac{A_{\mathrm{simplified}}}{A_{\mathrm{original}}}$$

Here, A denotes the convex hull area of the vein network relative to the design unit area. ACR characterizes the spatial footprint occupied by the vein network; a higher ACR indicates a larger spatial footprint, which may be associated with, but does not directly quantify, the actual textured contact area or contact-area fraction at the interface. The association observed between ACR and wet friction performance in this study should be read as an exploratory finding rather than as evidence of a confirmed causal mechanism.

### 2.6. Fabrication and Functional Validation of Tool-Grip Sleeves

The results reported in this section apply strictly to the present silicone material system, sample dimensions, and test parameters, and should not be generalized directly to industrial tool applications.

To evaluate the functional performance of each texture stage under wet-grip conditions, textures from Stages 1 through 3 were each fabricated into tool-grip sleeves for measurement of wet friction coefficient and directional recognition performance.

(1) Fabrication of grip sleeves

**Figure 4:**
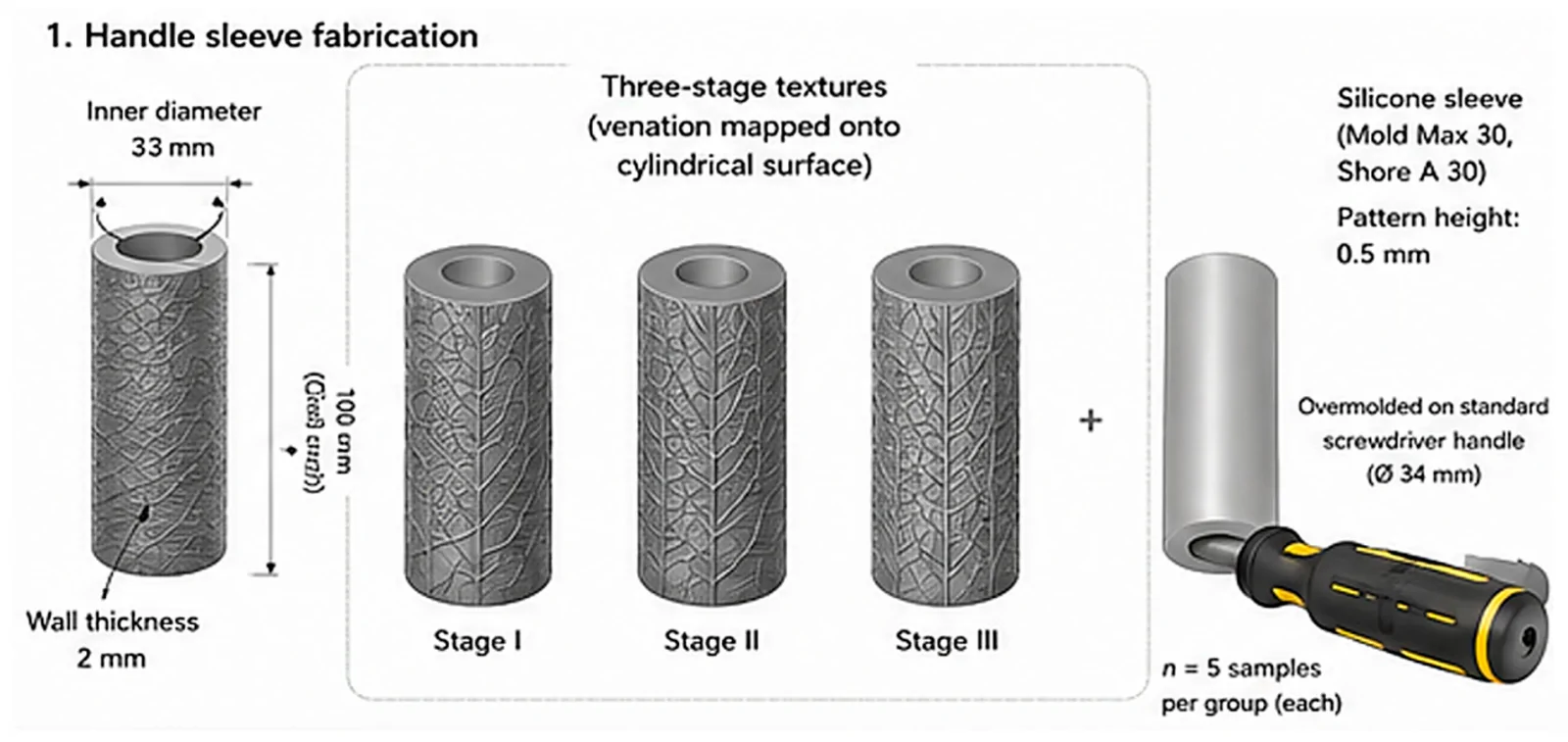
Fabrication of grip sleeves.

The vector networks for Stages 1, 2, and 3 were first laid out within a flat 60 × 60 mm design unit, then mapped onto cylindrical coordinates and cast in silicone elastomer (Mold Max 30, Smooth-On, Inc., Macungie, PA, USA; Shore hardness 30A) to form grip sleeves with a 33 mm internal diameter, 2 mm wall thickness, and a fixed textural relief height of 0.5 mm (refer to Figure 4). The flat unit dimensions (60 × 60 mm), once mapped onto the cylindrical geometry, cover the principal contact area of the sleeve’s grip section—the same prototype dimensions referenced in Section 4.4.

Fabrication used a commercial standard screwdriver handle (34 mm outer diameter) as the substrate, with the 33 mm inner diameter of the silicone sleeve achieving a secure fit through a 1 mm diametral interference (approximately 0.5 mm radial interference). Smooth, textureless silicone sleeves were also fabricated as controls, with five samples prepared per group.

(2) Wet friction coefficient test

Friction testing was designed with reference to the general principles of ASTM D1894-24 [25], Standard Test Method for Static and Kinetic Coefficients of Friction of Plastic Film and Sheeting. However, this test configuration deviates from the standard in the following respects: (1) the specimen was a cylindrical silicone grip sleeve rather than a flat plastic film; (2) the counter-material was fresh pigskin, used to approximate the tribological properties of human palm skin, rather than the material specified by the standard; (3) the interface included a thin water film to simulate moist-hand handling conditions. These deviations were intentional adaptations required to evaluate a curved hand-tool grip interface, reflecting the exploratory, application-oriented nature of this study; the protocol should accordingly be understood as adapted from, rather than strictly compliant with, ASTM D1894-24. The cylindrical texture mapping rule and test-direction definitions were as follows.

**Figure 5:**
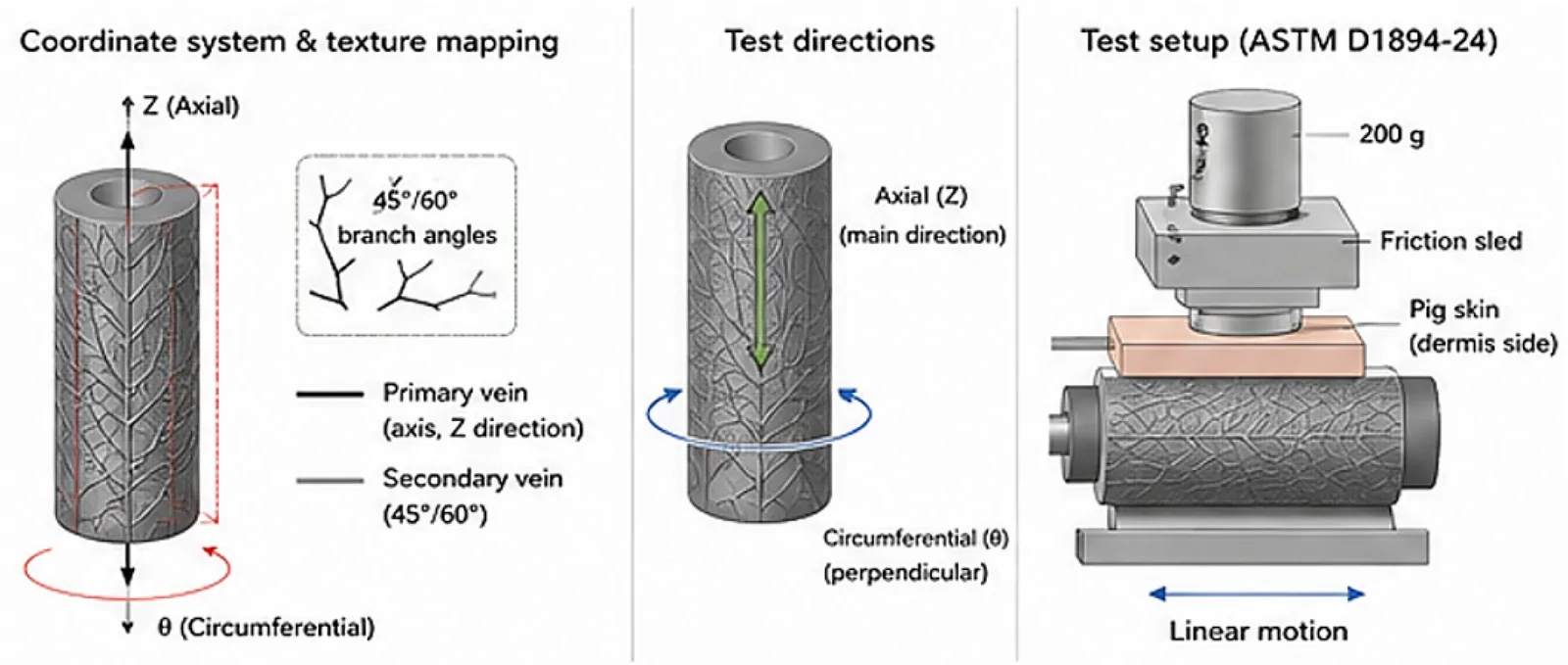
Wet friction coefficient testing (ASTM D1894-24).

1. Cylindrical texture mapping rule. The central axis (Z-axis) and circumferential direction (θ-axis) of the standard screwdriver handle’s grip section were used to establish a cylindrical coordinate system. The simplified venation patterns from Stages I–III were mapped onto this cylindrical surface, with the primary veins of the camphor leaf—used as the principal structural axis—arranged parallel to the handle’s axial direction (Z-axis). The angle between secondary and primary veins strictly followed the standardized 45°/60° branching angles defined in the simplification algorithm, distributed symmetrically along the circumferential direction (θ-axis). Textural relief height was uniformly set to 0.5 mm, consistent with the sleeve fabrication parameters (refer to Figure 5).
2. Definition of Test Direction. The primary (and only reported) test direction corresponds to the texture’s principal orientation—parallel to the handle axis (Z-axis) and aligned with the primary vein direction—perpendicular to the plane of the standardized 45°/60° secondary-vein branching arrangement. A circumferential (θ-axis) test configuration was considered during protocol design as a means of assessing potential frictional anisotropy, but was not implemented in the present study; the friction data reported below therefore reflect only the axial test direction.
3. Test parameters and quality control. Prior to testing, 0.2 mL of deionized water was sprayed evenly onto each grip sleeve surface to form a thin liquid film, simulating sweat or moisture during handling; fresh pigskin was used as the counter-material to approximate the tribological properties of human palm skin. The normal load was 200 g, sliding distance 50 mm, sliding speed 150 mm/min, with the test environment maintained at 25 °C and 50% relative humidity.

Five independent grip-sleeve samples were prepared for each experimental group, with each sample measured three times under identical test conditions; the three measurements were averaged to form a single statistical unit, and the reported kinetic friction coefficient reflects the mean ± SD across the five independent samples per group. Dry friction coefficients were measured under the same test direction and parameters.

(3) Blindfolded direction-recognition test

This experiment was designed to test whether geometrically regularized textures exhibit tactile anisotropy, rather than to directly assess anti-slip performance. An improvement in direction-recognition accuracy may suggest anisotropic frictional behavior, but the two are not equivalent; friction coefficient and direction-recognition accuracy are reported separately in this study, and the latter is not treated as a direct measure of anti-slip effectiveness.

The definition of the texture’s default primary direction was identical to the cylindrical mapping rule used in friction testing: the screwdriver handle’s central axis served as the axial reference (Z-axis), with the texture’s default primary direction aligned to the handle axis and to the principal venation direction of the biomimetic skeleton—perpendicular to the plane of the algorithm-regularized secondary-vein branching angles (45°/60°). The primary orientation of the texture was randomly assigned as 0° or 180° (axially upward or downward) across test samples, with sample counts balanced between the two orientations.

Twelve healthy adults (six male, six female, aged 22–35) were recruited, none with known tactile impairment of the hand or professional background in tool design; individuals with substantial prior knowledge of camphor leaf morphology were excluded. Two parallel conditions were tested: bare-handed (simulating everyday household tool use) and thin cotton-glove (simulating industrial maintenance and outdoor work scenarios), with identical procedures and parameters across both.

All testing was conducted in a quiet, temperature- and humidity-controlled room, with participants wearing opaque blindfolds throughout to eliminate visual input. Each participant completed three practice trials before formal testing to become familiar with the grip technique and minimize bias from unfamiliarity with the task. In the formal test, each participant evaluated all three texture stages (I, II, III), with ten trials per stage under each condition; trial sequence was randomized using a Latin square design to control for order and learning effects. In each trial, the experimenter presented a handle with a randomly assigned primary orientation; the participant gripped it in a standard screwdriver grip and, within ten seconds, made a forced-choice judgment of 0° or 180° based on tactile cues alone, with response accuracy recorded for each trial.

The primary outcome measure was each participant’s mean direction-recognition accuracy. Hand condition (bare-handed vs. cotton-gloved) was included as a secondary, practice-oriented factor reflecting two representative use scenarios, rather than as a primary target factor in the pre-specified analysis plan. The pre-specified main analysis compared direction-recognition accuracy across the three texture stages, with data from the two hand conditions pooled for this primary comparison; a formal test of the texture stage × hand condition interaction was not part of the original analysis plan for this exploratory study (see Section 4.4). Percentage data were arcsine square root-transformed prior to analysis to satisfy normality assumptions. Overall differences among the three texture stages were assessed by one-way repeated-measures ANOVA, followed by Tukey’s HSD post hoc test for pairwise comparisons, which controls the family-wise error rate via the studentized range distribution; significance was set at α = 0.05.

(4) Manufacturing fidelity inspection

Demolded, replicated textures were examined under microscopy to assess structural fidelity, with the minimum reproducible line width of the manufactured texture also measured.

### 2.7. Statistical Analysis

Sample size and statistical power: This study is exploratory and proof-of-concept in nature; sample size was determined primarily by experimental feasibility rather than by a priori formal power analysis. To evaluate the statistical power actually achieved, a post hoc power analysis was conducted using G*Power 3.1, based on a repeated-measures ANOVA (within-subjects factors) model at α = 0.05, using the observed effect size from the direction-recognition experiment (partial η^2^ = 0.84). The resulting power exceeded 0.95, indicating adequate sensitivity for detecting large within-subject effects. Given the exploratory scope of this study, however, this post hoc result only confirms the study’s capacity to detect large effect differences—it does not by itself constitute sufficient evidence of population-level effect stability.

Reliability verification: Cohen’s κ was used to assess inter-operator agreement in vein-layer classification (primary/secondary/tertiary) obtained from the two double-traced specimens. Geometric tracing accuracy (MNND and Hausdorff distance) was assessed separately as a descriptive measure of positional consistency between the two independently traced specimens, expressed as mean ± SD distance at branch junctions relative to a 0.5 mm tolerance threshold, rather than as a formal κ-based agreement statistic.

Comparison of simplification metrics across stages. CSR and ACR were compared using one-way repeated-measures ANOVA. Where overall differences reached statistical significance, Tukey’s HSD post hoc test was applied for pairwise comparisons, which controls the family-wise error rate via the studentized range distribution; effect sizes were reported as partial η^2^. Because the definition of TRI differs fundamentally in Stage 3 from the first two stages, TRI was treated as descriptive statistics only and excluded from the repeated-measures ANOVA.

Comparison of wet friction coefficients. Differences in the wet friction coefficient between the smooth control group and the three biomimetic texture groups were assessed by one-way ANOVA, with the mean of each independently fabricated specimen (*n* = 5) treated as the unit of statistical analysis. Normality was verified via the Shapiro–Wilk test and homogeneity of variance via Levene’s test prior to analysis; where overall differences were significant, Tukey’s HSD post hoc test was applied, which controls the family-wise error rate via the studentized range distribution, and effect sizes were likewise reported as partial η^2^.

Comparison of direction-discrimination accuracy. Percentage data were arcsine-transformed to satisfy normality assumptions, and differences among the three stages were tested via one-way repeated-measures ANOVA, with post hoc testing and multiple-comparison correction following the same procedure described above.

## 3. Results

### 3.1. Morphological Extraction and Tracing Accuracy

Manual layered tracing extracted venation structures from all 12 specimens, each exhibiting the basal actinodromous venation pattern characteristic of the species. Secondary vein counts ranged from 13 to 18 per leaf (mean 14.9 ± 1.9), and the original tracings averaged 3847 ± 512 total anchor points, of which tertiary venation accounted for roughly 89%. Qualitative inspection confirmed that the primary and secondary vein skeleton preserved the original leaf vein structure, supporting its use as a geometric prototype for anti-slip hand-tool grip texturing.

Inter-rater agreement was κ = 0.87 (95% CI: 0.81–0.93) for CAM-07 and κ = 0.84 (95% CI: 0.77–0.90) for CAM-11. This range of 0.84–0.87 falls within the “almost perfect” category on the Landis and Koch scale (0.81–1.00) [26], indicating that the tracing protocol is reproducible at this sample size.

Geometric accuracy was likewise high: the mean nearest-neighbor distance (MNND) at branch junctions was 0.27 ± 0.09 mm for one specimen and 0.31 ± 0.11 mm for the other, both well within the 0.5 mm tolerance threshold, with corresponding Hausdorff distances of 0.42 mm and 0.45 mm.

### 3.2. Visualized Output of the Three-Stage Simplification

Stage 1 reduced the anchor count from roughly 3847 to about 420 (CSR ≈ 0.11), with TRI = 1.00, producing a high-density network. Stage 2 compressed the anchor count further, to approximately 180 (CSR ≈ 0.05), with TRI at 0.96 ± 0.03. Curved secondary veins were replaced with straight lines or circular arcs, and secondary-vein branching angles were normalized toward the 45°/60° targets within a ±5° tolerance. The actual post-processing coverage and mean deviation from target depend on the simplification algorithm’s output across the full dataset and are treated as an internal step in algorithm optimization rather than incorporated into the final reported metrics. The 0.04 decline in TRI, from 1.00 to 0.96, stems from the removal of very short vein segments (<2 mm, median 1 per specimen). This stage’s output is the regularized network. Stage 3 retained only the three primary veins (CSR ≈ 0.01, anchor count 24–36), with TRI = 0.33 ± 0.05. As defined in Section 2.5, this value reflects the residual positional information of branch nodes along the primary-vein skeleton—a different quantity from TRI in the first two stages, which reflects the completeness of branching connectivity—and cross-stage comparison is therefore not appropriate. This stage’s output is a minimalist skeleton, whose functional performance at scales below 10 mm has not been experimentally verified.

### 3.3. Results of the Quantitative Assessment of Simplification Degree

Table 2 summarizes the mean values of the three metrics across all 12 specimens, providing the quantitative basis for selecting among anti-slip texture options for hand-tool grips. Stage 1 achieved the highest ACR (0.97 ± 0.02) and the most pronounced anti-slip performance; Stage 2’s CSR sat at an intermediate level (0.05 ± 0.01), balancing anti-slip function against manufacturing cost; and Stage 3’s CSR was lowest (≈0.01), with manufacturing complexity correspondingly minimized.

**Figure 6:**
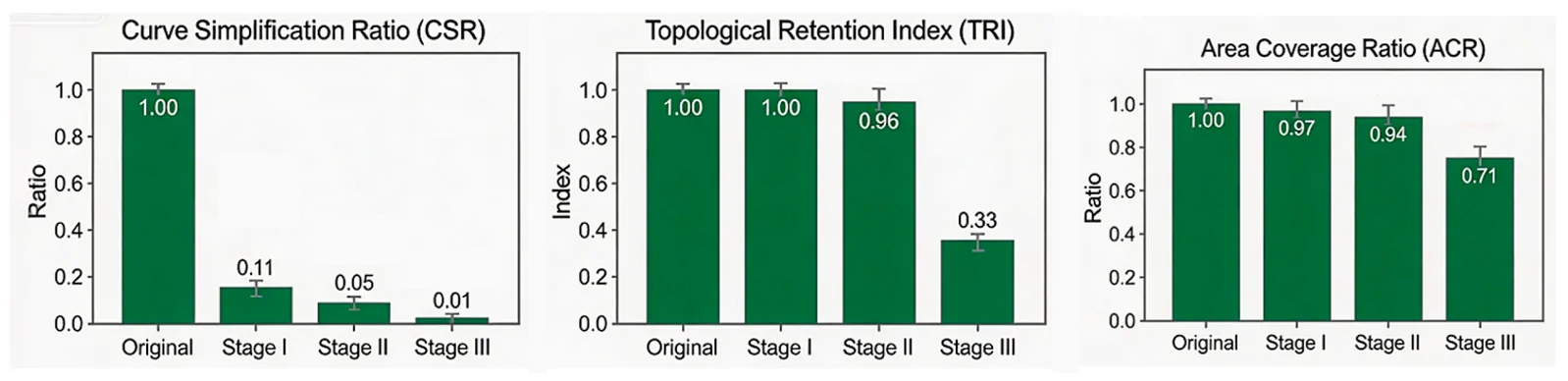
Statistical results of the three quantitative metrics across the three stages.

Repeated-measures ANOVA revealed statistically significant differences across the three stages for both CSR and ACR. With degrees of freedom of (2, 22) in both cases, between-stage differences reached significance for CSR (F = 156.3, *p* < 0.001, partial η^2^ = 0.93) and ACR (F = 78.5, *p* < 0.001, partial η^2^ = 0.88). Because Stage 3’s TRI is computed on a different basis from the first two stages, it was treated purely as a descriptive reference and excluded from the cross-stage ANOVA. Tukey’s HSD pairwise comparisons confirmed that all three pairwise combinations (Stage 1 vs. Stage 2, Stage 2 vs. Stage 3, Stage 1 vs. Stage 3) were statistically significant for both CSR and ACR (all *p* < 0.001). The three simplification stages are thus clearly distinguishable in both degree of simplification and geometric character (refer to Figure 6), giving designers a basis for selecting the appropriate texture according to a grip’s specific functional requirements.

### 3.4. Validation Results from the Grip-Sleeve Carrier

To evaluate the functional performance of the three-stage textures in a wet-grip interface, this study fabricated each stage’s texture into a silicone tool-grip sleeve and subjected all sleeves to wet friction and tactile direction-discrimination testing under identical material, surface dimension, and load parameters.

Wet friction results were based on the mean of independently fabricated samples within each group (*n* = 5). A significant difference emerged between the smooth control group and the three biomimetic texture groups (F(3,16) = 84.27, *p* < 0.001), with the control showing the lowest mean wet friction coefficient (0.21 ± 0.03). Stage 1 texture produced the highest wet friction coefficient (0.82 ± 0.05), followed by Stage 2 (0.67 ± 0.04) and then Stage 3, which declined further to 0.49 ± 0.05. Retention of higher-order branching structure tracked closely with enhanced wet friction response; as textural complexity decreased, the friction-enhancing effect weakened correspondingly.

**Figure 7:**
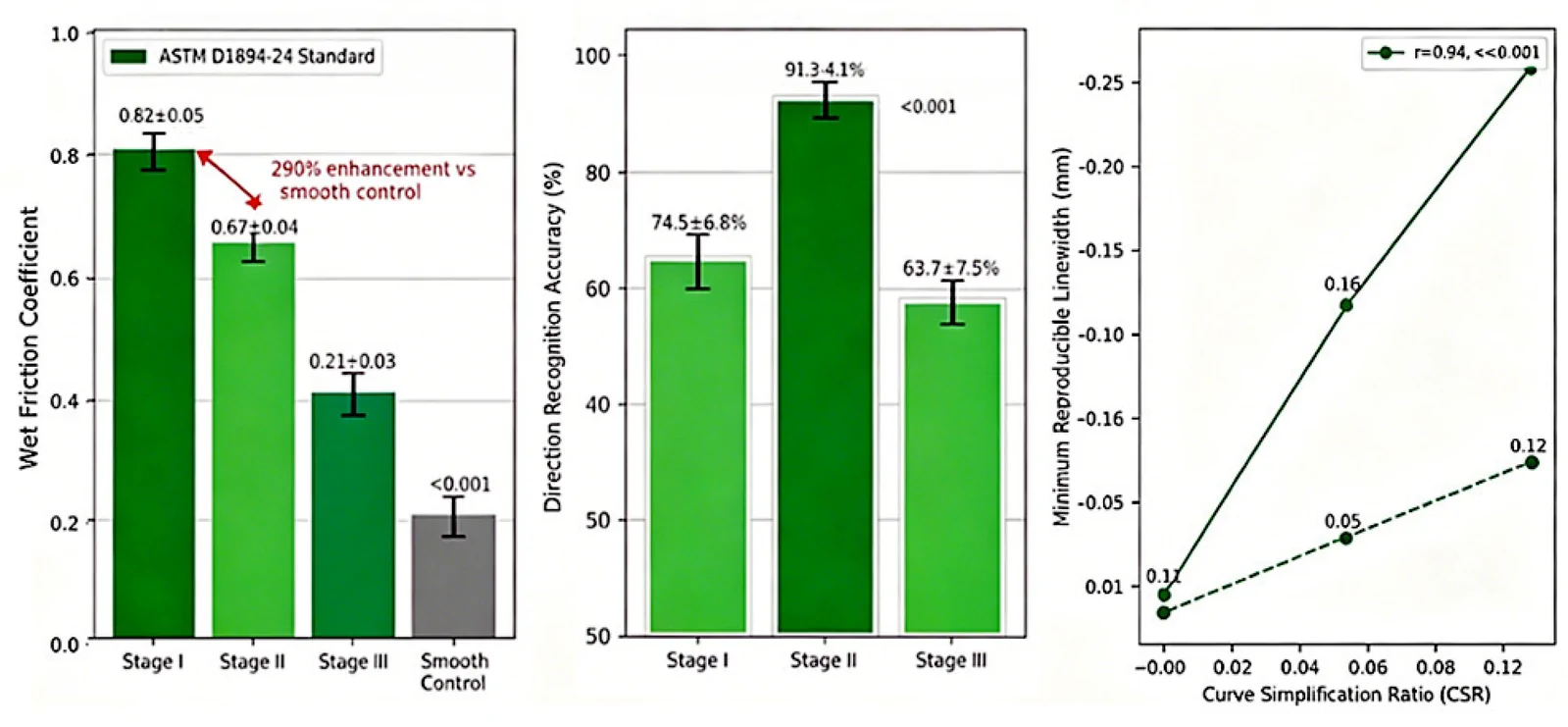
Summary of functional validation results across the three texture stages.

Tactile direction-discrimination testing likewise showed significant differences across stages (F(2,22) = 56.18, *p* < 0.001). Stage 2 achieved the highest direction-discrimination accuracy (91.3% ± 4.1%), significantly outperforming both Stage 1 (74.5% ± 6.8%) and Stage 3 (63.7% ± 7.5%). Moderate geometric regularization appears to strengthen the consistency of directional tactile signals, improving participants’ recognition ability; by contrast, the high local detail complexity of Stage 1 introduced randomness into tactile stimulation, while the excessive structural simplification of Stage 3 weakened the discriminability of directional features (refer to Figure 7).

On the manufacturing side, Stage 1 texture showed minor demolding adhesion in locally high-density regions. Stage 2 reliably reproduced a 0.16 mm line width with good boundary integrity, while Stage 3—the least demanding to manufacture—faithfully reproduced a 0.12 mm line width.

This study did not include a univariate control design, so the observed functional differences cannot be attributed to any single geometric parameter: curvature, branching angle, vein segment length, local density, and topological complexity all varied simultaneously across stages. These results indicate only an association between overall geometric regularization and enhanced wet friction and directional tactile perception; a strict causal relationship cannot be established directly from the current data.

The silicone coating material, porcine-skin friction medium, and sample dimensions used in this study were all set under proof-of-concept conditions. The results therefore serve as preliminary evidence for the feasibility of biomimetic grip texturing and should not yet be extrapolated directly to real-world industrial tool contexts.

## 4. Discussion

### 4.1. Rationale for Manual Tracing

Manual tracing was chosen over automated segmentation because the design workflow tolerates neither incomplete vector paths nor ambiguous hierarchical representation. Existing tools—ImageJ vein-analysis plugins, deep-learning segmentation networks—are optimized to extract statistical descriptors of venation (vein length density, mesh area, and the like) rather than to generate clean vector paths ready for direct design use. Artifacts common in segmentation, such as discontinuous vein segments and spurious connections, barely affect statistical output, yet they render a vector graphic unusable for texture design and manufacturing outright.

In preliminary tests, six camphor leaf images processed through adaptive thresholding and binary skeletonization showed discontinuities in 34.7% of secondary vein segments and spurious connections in 21.3% of tertiary segments. Correcting these artifacts to a standard suitable for grip-texture design took an average of 2.5 h of manual post-processing per specimen—well beyond the 1.8 h per specimen required for manual tracing in this study.

Manual tracing externalizes and standardizes judgments of visual salience: the operator can directly assess how much a given vein segment contributes to recognizable camphor-leaf character or to grip anti-slip function, and this judgment process remains traceable. The high κ values obtained from the two double-traced specimens (0.87 and 0.84), together with the geometric fidelity results, confirm that the layered tracing protocol developed here achieves reliable reproducibility across operators. Two specimens is admittedly a limited basis for validation, and the result should be read as a preliminary proof of concept rather than a settled reliability claim. Establishing a generalizable, standardized tracing protocol would require independent double-tracing of at least 10–15 specimens and calculation of an intraclass correlation coefficient (ICC); the κ values reported here are accordingly preliminary evidence, not a mature reliability metric. A semi-automated pipeline—U-Net pre-segmentation followed by manual refinement—also merits exploration for larger-scale future applications.

### 4.2. Morphological Lineage Constructed by the Three-Stage Simplification

The three-stage algorithm yields a quantifiable morphological lineage: Stage 1 produces a high-density network, Stage 2 a regularized network, and Stage 3 a minimalist skeleton. Where prior biomimetic texture research has largely relied on a single regular geometric pattern, this study’s staged, lineage-based output lets designers select the abstraction level suited to a given application—anti-slip demand, tactile-recognition precision, manufacturing cost—directly from a continuous spectrum, replacing the trial-and-error design mode typical of earlier approaches. Each stage is defined by explicit geometric operations and paired with the CSR and ACR metrics, together forming a structured decision framework. Stage 3’s suitability at scales below 10 mm remains experimentally unverified.

The 45° and 60° branching-angle regularization targets were established from empirical statistics on nine specimens, and the ±5° tolerance was set through iterative pilot testing. This tolerance draws a quantitative boundary between geometric regularization—constrained by manufacturing feasibility (the 2 mm vein-segment threshold, the 2:1 line-width ratio)—and morphological naturalness, constrained by the need to preserve species-specific character. Gestalt principles of perceptual organization offer a theoretical basis for why such regularization improves textural legibility [27]. The direction-recognition experiment confirms this improvement along the tactile dimension: accuracy rose from 74.5% at Stage 1 to 91.3% at Stage 2, indicating that moderate geometric regularization does not compromise the texture’s functional discriminability.

Stage 3’s TRI of 0.33 ± 0.05 reflects the residual topological information at that stage, but because its statistical basis differs from that of Stages 1 and 2, the value should not be compared directly across stages, nor interpreted as a proportional loss of topological information; its role here is purely as a descriptive reference for that stage, used alongside CSR and ACR. Ronellenfitsch and Katifori [28], working from a network-elasticity optimization perspective, argue that the skeletal structure of biological transport networks trades mechanical performance against geometric parsimony—a framing that resonates with this study’s Stage 3 finding: a texture retaining only the primary-vein skeleton still exhibits measurable friction and tactile function, echoing the broader theme of structural simplification alongside functional retention. The simplification algorithm itself draws on morphogenetic modeling of plant venation [29]; where the original algorithm simulates vein development, this study runs it in reverse—toward simplification and lineage construction—constituting, methodologically, a repurposing from “generation” to “reduction.”

### 4.3. Functional Translation of the Tool Grip Under Wet Anti-Slip Conditions

To validate this functional translation, the three-stage textures were fabricated as silicone grip sleeves and tested for both wet friction coefficient and directional tactile recognition. The results show that different degrees of geometric simplification alter the texture’s interfacial contact characteristics, and functional differences follow from that shift.

Stage 1, which retains the secondary vein network, produced the highest wet friction coefficient (0.82 ± 0.05), exceeding both Stage 2 (0.67 ± 0.04) and Stage 3 (0.49 ± 0.05). This study did not, however, undertake contact-mechanics or fluid-dynamics analysis, so the pattern cannot be attributed to any specific geometric parameter—texture density, local curvature, or topological complexity among them.

**Figure 8:**
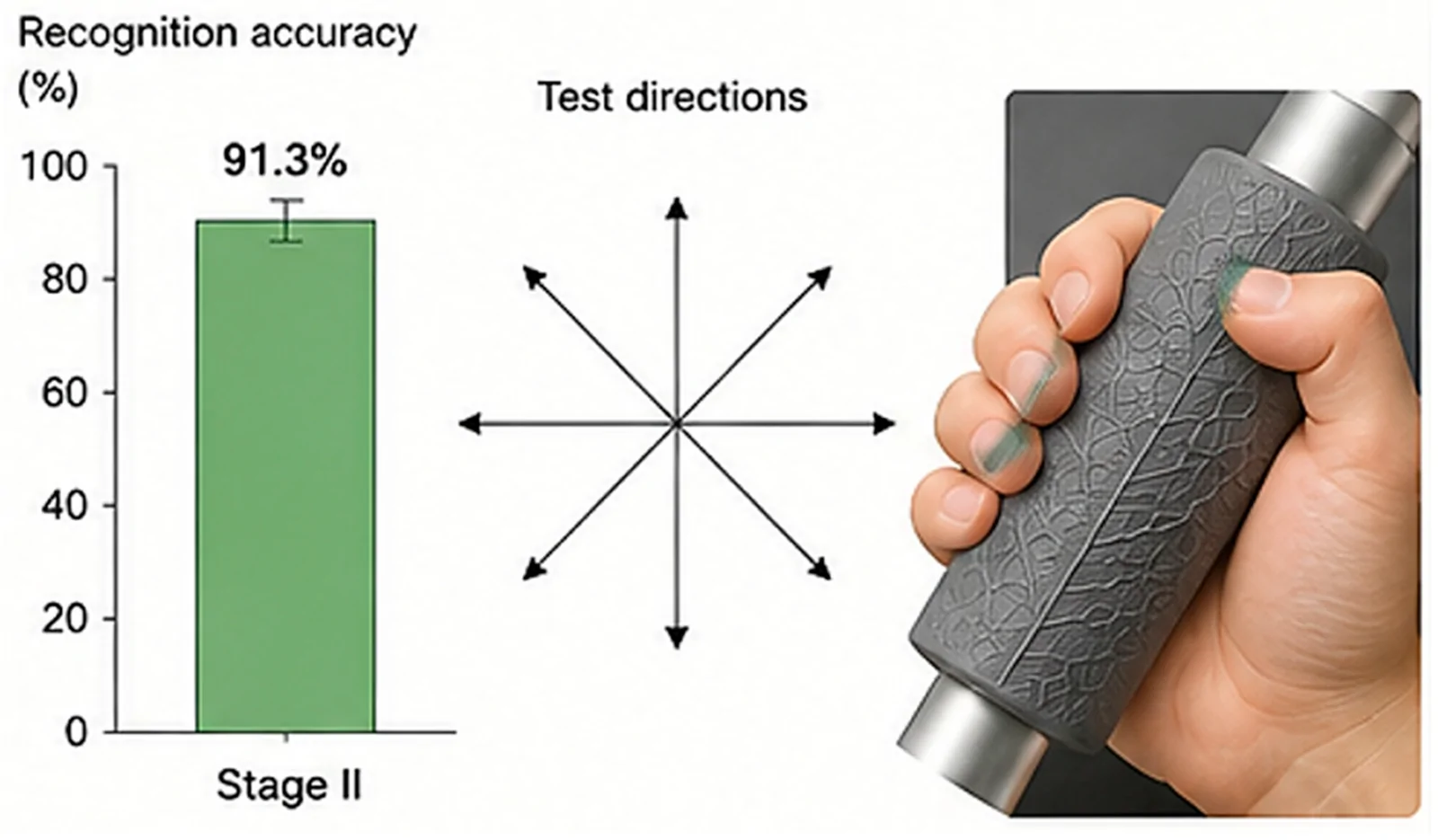
Directional recognition accuracy at Stage 2.

Stage 2 combined a wet friction coefficient of 0.67 ± 0.04, direction-recognition accuracy of 91.3% ± 4.1%, and a line width of 0.16 mm (refer to Figure 8). The direction-recognition results suggest that moderate geometric regularization may have improved the consistency of directional tactile signals. Because the simplification process altered curvature, branching angle, vein-segment length, and local density simultaneously, though, the current data cannot establish a strict causal link between any single geometric variable and the observed functional improvement.

Stage 3 was the least demanding to manufacture, with a minimum line width of 0.12 mm—but its wet friction response and direction-recognition accuracy both declined markedly, suggesting that excessive simplification may erode the tactile and frictional richness of the textured interface.

The wet friction measurements in this study were obtained only in the axial test direction; a circumferential configuration was considered during protocol design but not implemented. Consequently, whether the regularized venation texture exhibits direction-dependent (anisotropic) friction behavior was not evaluated here and remains an open question for future work using a complete axial/circumferential friction protocol.

The core aim of this study is to test the feasibility of the design pathway “venation geometric abstraction → parametric regularization → functional texture generation,” not to build a complete engineering tribological model. The present findings constitute exploratory design evidence, not a finalized optimization scheme for industrial tool interfaces. Where prior biomimetic texture research has typically followed a one-way “generate texture → test performance” pipeline, this study offers an iterative path—“design parameters → performance feedback → parameter adjustment.” Future work drawing on univariate control experiments, contact-pressure analysis of the human hand, CFD simulation of liquid films, and surface tribological modeling could progressively clarify the relationship between specific geometric parameters and functional outcomes, building toward a more rigorous mechanistic framework.

### 4.4. Limitations and Future Directions

This study is subject to several limitations.

First, specimen collection was geographically restricted to three old-growth camphor stands in Jiangxi Province. Wan et al.’s comparison of fruit phenotypic traits across eleven camphor provenances found a mean differentiation coefficient as high as 80.1% [22]—implying that broader geographic sampling will be needed in future work to confirm the generalizability of this abstraction framework across camphor specimens of different origin.

Second, while the two double-traced specimens established inter-rater reliability (κ ≥ 0.84) and geometric fidelity, the sample remains small; a larger double-tracing dataset is a necessary next step.

Third, Stage 3’s functional performance at smaller scales lacks direct verification. The prototype dimensions used here (60 × 60 mm, with a texture relief height of 0.5 mm) have not been tested experimentally below a 10 × 10 mm scale or at finer line widths.

Fourth, no physical model yet links texture geometric parameters to friction performance; the current findings sit closer to empirical validation than mechanistic explanation.

Fifth, manufacturing feasibility—and the relationship between CSR and that feasibility—has been examined only under silicone-molding conditions; broader industrial processes such as injection molding or laser engraving remain untested.

Sixth, this workflow was designed specifically for the three-veined (actinodromous) structure of camphor leaves; other venation types—pinnate, palmate, and parallel—have not yet been validated within this framework.

Seventh, TRI’s statistical basis at Stage 3 differs from that of the first two stages, limiting its comparability across all three stages; future work might redefine TRI to maintain a consistent statistical basis throughout.

Eighth, the direction-recognition experiment included both bare-handed and cotton-gloved conditions to reflect practical use scenarios, but the primary analysis pooled data across these two conditions and did not formally test the texture stage × hand condition interaction. Whether hand condition moderates the effect of texture stage remains an open question that merits dedicated investigation in future studies with larger sample sizes.

Ninth, continuous geometric agreement (MNND and Hausdorff distance) was assessed descriptively (mean ± SD distance relative to a 0.5 mm tolerance threshold) based on two double-traced specimens, rather than through a formal reliability coefficient such as ICC. Future studies with larger sample sizes and formal ICC computation would strengthen the quantitative assessment of tracing reproducibility.

Tenth, wet friction coefficient measurements in this study were conducted only in the axial test direction. Directional friction behavior (axial vs. circumferential) was not evaluated; systematic characterization of directional friction is listed as a priority for future work.

Future research might proceed along several lines: (1) extending the current framework to additional venation types; (2) expanding the sample size and age range for double-tracing and tactile-perception experiments; (3) testing Stage 3 texture performance at 10 mm and 20 mm scales; (4) developing a semi-automated tracing tool with CSR and TRI as adjustable parameters; and (5) exploring applications of simplified venation networks in computational fluid dynamics and lightweight structural mesh generation. Vein growth processes have already been applied to topology optimization of surface support structures [30], offering a useful reference point for this last direction.

## 5. Conclusions

This study establishes a parametric biomimetic design workflow that transforms the venation structure of *Cinnamomum camphora* leaves into functional surface texture, validated as a proof of concept through wet-grip experiments. The three proposed simplification metrics—CSR, TRI, and ACR—offer a quantifiable basis for weighing texture complexity, topological fidelity, and manufacturing feasibility against one another.

(1) A standardized protocol for specimen collection, imaging, and layered tracing reliably extracted the hierarchical structural features of camphor leaf venation, with inter-rater reliability preliminarily confirmed.
(2) The three-stage geometric simplification algorithm constructs a morphological lineage spanning from a high-density network to a minimalist skeleton; cross-stage comparison of CSR and ACR, together with the descriptive reference value of TRI, demonstrates a quantitative correspondence among texture complexity, topological character, and manufacturing difficulty.
(3) Textures at different stages differed in wet friction coefficient (0.82 ± 0.05 for Stage 1, 0.67 ± 0.04 for Stage 2, 0.49 ± 0.05 for Stage 3) and direction-recognition accuracy (74.5% ± 6.8% for Stage 1, 91.3% ± 4.1% for Stage 2, 63.7% ± 7.5% for Stage 3). The functional mechanism underlying Stage 2’s performance warrants further verification through human-factors experiments, contact-mechanics analysis, and fluid-dynamics simulation.

This study does not propose a ready-to-deploy industrial solution. Rather, it establishes a reproducible proof-of-concept pathway linking biological venation structure, geometric abstraction, parametric simplification, and functional texture validation—one that may inform future work on biomimetic grip surfaces, directional tactile interfaces, and functional surface texture design more broadly. The goal of this study is not to build a comprehensive tribological theory, but to test the feasibility of the design pathway “venation geometric abstraction → parametric regularization → functional texture generation.” As a design-driven proof of concept in biomimicry, this work is distinct in character from conventional engineering tribology research.

## Figures and Tables

**Table 1:** Summary of the three-stage geometric simplification algorithm.

Stage	Layers Retained	Geometric Operations	Output Characteristics	Application
Stage 1: Structural Preservation	Primary + secondary vein	No geometric alteration; tertiary vein layer removed only	High-density network, natural curvature retained	High anti-slip demand (pliers, pruning shears, etc.)
Stage 2: Geometric Regularization	Primary + regularized secondary veins	Chord-height ratio evaluation (threshold 0.05); pronounced curvature approximated by bi-tangent circular arcs, otherwise straightened; branching angles regularized to 45°/60° (±5° tolerance); vein segment length normalized (mean ±10%); segments shorter than 2 mm removed; primary-to-secondary line-width ratio of 2:1	Regularized network, balancing anti-slip performance and comfort	Conventional hand-tool grips
Stage 3: Feature Extraction	Primary veins only	Secondary and higher-order veins removed; three primary veins redrawn at a uniform 1.0 pt line width	Minimalist skeleton, low-density directional texture	Manufacturing-simplicity-priority scenarios

Note: The basis for the key parameters at each stage—chord-height ratio threshold of 0.05, angular tolerance of ±5°, and vein segment length threshold of 2 mm—is detailed in (1)–(3) above. The branch nodes retained in Stage 3 correspond to roughly one-third of the original total, giving TRI ≈ 0.33.

**Table 2:** Quantitative evaluation of simplification (mean ± standard deviation, *n* = 12).

Simplification Stage	CSR	TRI	ACR
Original (full tracing)	1.00 (References)	1.00 (References)	1.00 (References)
Stage 1 (structural preservation)	0.11 ± 0.02	1.00 ± 0.00	0.97 ± 0.02
Stage 2 (geometric regularization)	0.05 ± 0.01	0.96 ± 0.03	0.94 ± 0.03
Stage 3 (feature extraction)	0.01 ± 0.00	0.33 ± 0.05	0.71 ± 0.08

## Data Availability

Additional data will be provided upon request.
